# Colonic Paneth cell metaplasia is pre-neoplastic condition of colonic cancer or not?

**DOI:** 10.1186/1477-3163-4-5

**Published:** 2005-02-12

**Authors:** Ryo Wada, Toshikazu Yamaguchi, Kenichi Tadokoro

**Affiliations:** 1 The Department of Pathology, Juntendo Izunagaoka Hospital of Juntendo University School of Medicine, Shizuoka, Japan; 2 The Department of Pathology(I), Juntendo University School of Medicine, Tokyo, Japan; 3 R & D Center, Biomedical Laboratories, Inc, Kawagoe, Japan

**Keywords:** Paneth cell metaplasia, K-ras mutation, loss of heterozygosity of microsatellite marker, colorectal cancer, microdissection

## Abstract

**Background:**

The carcinogenesis of colorectal cancer has been accepted by a model for a cascade of genetic alterations, named the adenoma-carcinoma sequence. In order to elucidate the carcinogenesis of the colorectal cancer more clearly, the genetic abnormalies of the non-neoplastic mucosal epithelium of the colon and rectum should be investigated. It has been speculated that colonic Paneth cell metaplasia (PaM) is one of the pre-neoplastic mucosa of colonic cancer. Therefore, we studied the propria mucosa of the right colon with PaM from the standpoints of the frequency of the K-ras codon 12 mutations (K-ras), which is initial genetic abnormality in colorectal cancer, and the loss of heterozygosity of microsatellite markers (LOH-MS), which has a relationship to development of colorectal cancer.

**Methods:**

Fifty-two regions with PaM histopathologically from 12 surgically resected right colon specimens were studied. DNA extraction of the colonic mucosa with PaM was obtained using a microdissection method, and the frequency of the K-ras of PaM was investigated by enriched polymerase chain reaction-enzyme linked mini-sequence assay, and the frequency of the LOH-MS (D2S123, D17S250 and D5S346) of PaM was examined by high resolution fluorescenced labeled PCR primers.

**Results:**

K-ras mutation was detected in fifteen regions among 52 PaM (28.9%). All mutations were a single mutation and GGT changed to AGT in eleven and GAT in four. LOH-MS were detected in twenty-one regions among 52 PaM (40.4%) (D2S123: 35.4%, 17/48 regions, D17S250: 13.7%, 7/51 regions, and D5S346: 0%, 0/52 regions). No K-ras mutations and LOH-MS were detected in the controls (Colorectal mucosa with no PaM).

**Conclusions:**

Colonic mucosa with Paneth cell metaplasia may be one of the pre-neoplastic mucosa in the development of the colonic epithelial neoplasia.

## Background

Vogelstein et al. [1] have reported the involvement of multistage genetic abnormalities in the development of colorectal cancers, and pointed out that K-ras mutation is the initial genetic abnormality in the adenoma-carcinoma sequence [2] of the development of the colorectal cancer. Recently, some reports have pointed out that the replication errors and loss of heterozygosity of the microsatellite markers have the development of the colorectal cancer [3,4].

However, the genetic abnormalities of the non-neoplastic mucosal epithelium of the colon and rectum has not been investigated, except the aberrant crypt foci [5] and hyperplastic polyp [6], although the colorectal epithelial neoplasia is derived from the colorectal mucosal epithelium. For the preventive medicine, the genetic abnormalities of the pre-neoplastic mucosa of the colorectal cancer should be known.

We have speculated previously that the colorectal Paneth cell metaplasia (PaM) is one of pre-neoplastic mucosa on the development of the colorectal epithelial neoplasias [7], because PaM were seen very frequently in the adjacent mucosa to the minute-sized colorectal epithelial neoplasias as well as within these neoplasias.

The main purpose of the present study was to investigate the frequencies of the K-ras codon 12 mutations (K-ras) and the loss of heterozygosity of dinucleotide microsatellite markers (LOH-MS) in the propria mucosa with PaM of the right colon.

## Methods

The materials were 12 surgically resected specimens of right colon, which had the colonic carcinomas were present, and histological diagnosis was assessed at the Department of Pathology, Juntendo Izunagaoka Hospital. Although we have intended to investigate the PaM in the normal colon of the individuals without cancer, using the biopsies specimens, it was difficult to detect the PaM in these specimens. No inflammatory bowel diseases were included in the materials. Informed consent was obtained from all the patients to investigate the genetic alterations in the current study.

The specimens were fixed in 10 % buffered formalin solution and prepared by cutting the non-neoplasitic area into 3 – 5 mm sections. Each section was embedded in paraffin and stained with hematoxylin and eosin (HE), followed by immunohistochemical staining for anti-lysozyme (DAKO, Japan). Immunohistochemical stainings were performed by the avidin-biotin-peroxidase-complex method, at dilution of 1 : 100.

Fifty-two colonic mucosal regions with PaM, distant from neoplastic lesion, aberrant crypt foci and hyperplastic polyp, were detected by HE staining and anti-lysozyme antibody staining, and these mucosa were used as the target regions in the current study.

All paraffin blocks of the target regions and 12 paraffin blocks with no PaM as controls of each materials were used for the DNA extraction.

### DNA extraction

Paraffin blocks with the target foci mentioned above were prepared for DNA extraction. The target foci were microdissected using a 20-gauge needle, comparing the slide with HE staining in the same position. The extracted DNA was diluted with 5 ml of TaKaRa DEXPAT (for DNA Extraction from Paraffin-embedded Tissue, TaKaRa Biomedical Inc.).

### Analysis of the K-ras

Mutation of K-ras was analyzed and compared by enriched polymerase chain reaction-enzyme linked mini-sequence assay (PCR-ELMA). In PCR-ELMA [8,9], upstream primer for the first and second PCR was 5'-TAAACTTGTGGTAGTTGG-AACT-3', downstream primer for the first PCR was 5'-GTTGGATCA-TATTCGTC-CAC-3', and downstream for primer the second PCR was 5'-CAAATGAT-CTGA-ATTAGCTG-3'. The first PCR reaction was performed containing 1 μL of DNA lysate, 100 μM dNTP, 1.5 mM MgCl_2_, 1 μM each primer, 0.625 U Taq DNA polymerase and 1 × PCR buffer [containing 10 mM Tris-HCl(pH 8.3 at 25 degrees of temperature), 50 mM KCl and 0.001%(w/v) gelatin] in a thermal cycler. Then, 10 μL of the denatured second PCR product was hybridized with probes to detect the K-ras codon 12 wild-type (GGT) and six mutant (GAT, GCT, GTT, AGT, CGT and TGT) DNAs were immobilized, at 55 degrees of temperature for 30 minutes, and 100 μL of biotinylated A and 0.01 U of TdqDNA polypmerase were added and incubation was continued at 55 degrees of temperature for 30 minutes.

**Figure 1:**
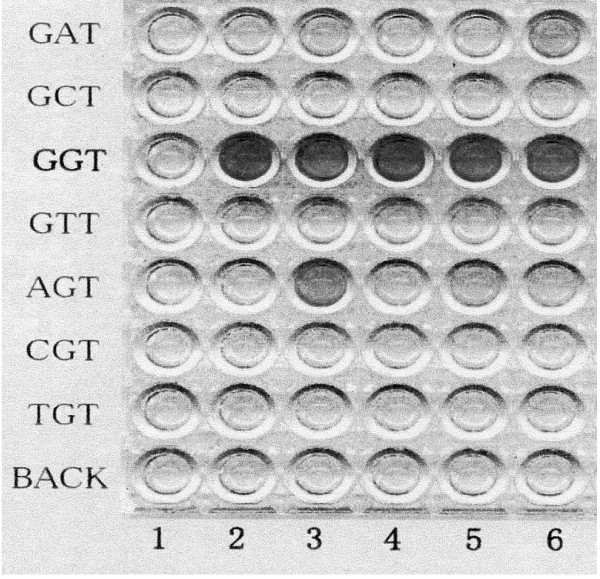
Enriched polymerase chain reaction-enzyme linked mini-sequence assay (PCR-ELMA), showing K-ras codon 12 mutation in the colonic Paneth cell metaplasia. AGT-type mutation was seen in line 3.

For development, 100 μL of avidin-horseradish peroxidase conjugate was added and the reaction was performed at room temperature for 30 minutes. Then, 100 μL of tetramethyl-benzidine (TMB) substrate was added and the plates were left to develop in the dark at room temperature for 20 minutes. Finally, 100 μL of stop solution was contained and the light absorbance of each sample was measured by spectrophotometry (Multiskan Multisoft, Labsystems, Tokyo) with a 450 nm filter wavelength (Figure 1).

### Analysis of the LOH-MS

Although we have intended to investigate the frequency of microsatellite instabilities using five microsatellite markers, which has been recommended by National Cancer Institute[10], the replication error or microsatellite instabilities could not be detected. Although the true reasons for these failures was not know, we may think that the target foci were too small and post-formalin solution condition with long time.

Therefore, only LOH was investigated described below. Three dinucleotide microsatellite markers (D2S123, D17S250 and D5S346) were selected for LOH among the microsatellite markers, recommended by National Cancer Institute, because commonly it has been thought to be difficult to study the LOH using mononucleotide microsatellite markers.

These LOH-MS were investigated using high resolution fluorescenced labeled PCR primers in proportion to the method of Tsuchida et al. [11]. The outline of it : 1. PCR was performed containing 1 μL of DNA lysate, 100 μM dNTP, 1.5 mM MgCl_2_, 1 μM each primer marked with fluorescent dye of three colors as blue, green and yellow, 0.625 U Taq DNA polymerase and 1 × PCR buffer [containing 10 mM Tris-HCl(pH 8.3 at 25°C), 50 mM KCl and 0.001%(w/v) gelatin] in a thermal cycler. 2. The electrophoresis was conducted 2 hours by means of an ABI-377 DNA auto-sequencer (PE Biosystems, Inc., Foster City, CA, USA). 3. A comparison was made of peaks of same marker arising from normal tissue and the propria mucosa with PaM, using the Gene Scan TM waveform analyzed softwave, and LOH were assessed. That is to say, LOH (+) was assessed when the ratio of the peak area of (the propria mucosa with PaM/normal tissue) was less than 70 % or was more than 143 %.

The data were analyzed statistically with Student's t-test (t-test) and chi-square test ; a p-value of less than 0.05 was considered to be significant.

## Results (Table 1)

**Table 1:** K-ras mutation and the loss of heterozygosity of microsatellitemarkers of colonic Paneth cell metaplasia

	K-ras*	D2S123	D17S250	D5S346	Total LOH*
PaM	28.9%	35.4%	13.7%	0%	40.4%
	(15/52)	(17/48)	(7/51)	(0/52)	(21/52)

Normal	0%	0%	0%	0%	0%
	(0/8)	(0/8)	(0/8)	(0/8)	(0/8)

PaM : Colonic Paneth cell metaplasia,

Normal : Normal colonic mucosa, K-ras : K-ras codon 12 mutation,

* : between PaM and Normal, p < 0.01, chi-square test

K-ras mutation was detected in fifteen regions among 52 PaM (28.9 %). All mutations were a single mutation. For K-ras mutation patterns, 11 showed GGT to AGT, and four showed to GAT.

LOH-MS was detected in twenty-one regions among 52 PaM (40.4 %) (D2S123: 35.4 %, 17/48 regions, D17S250: 13.7 %, 7/51 regions, and D5S346: 0 %, 0/52 regions). No K-ras mutations and LOH-MS were detected in the controls (Colorectal mucosa with no PaM, no neoplastic lesion, no aberrant crypt foci and no hyperplastic polyp).

Thus, the frequency of both K-ras mutation and LOH-MS in the colonic mucosa with PaM were significantly higher than those of the controls (p < 0.01, chi-square test).

## Discussion

K-ras mutations have been detected in several human neoplasias [12-14], and it has been pointed out that K-ras mutation is the initial genetic abnormality in the development of the colorectal cancers [1]. Recently, the replication errors of the gene is thought to be important in the development of the colorectal cancer. And the loss of heterozygosity of the microsatellite markers, which are often used as the targets in the investigation for the replication errors of the gene, is also considered to be important in the development of the colorectal cancer [3,4]. Namely, the carcinogenesis of colorectal cancer is almost clarified.

In order to conclude the carcinogenesis of the colorectal cancer more clearly, the genetic abnormality of the non-neoplastic mucosal epithelium of the colon and rectum should be investigated, however, because the colorectal cancers are derived from the colorectal mucosa. It is also important for the preventive medicine of the colorectal cancer to know the carcinogenesis of it.

However, until now, the genetic abnormalities of the colorectal non-neoplastic mucosa is unclear, except the aberrant crypt foci [5] and hyperplastic polyp [6].

We have already reported that the colorectal Paneth cell metaplasia (PaM) is one of pre-neoplastic mucosa on the development of the colorectal epithelial neoplasias [7], because PaM were seen very frequently in the adjacent mucosa to the minute-sized colorectal epithelial neoplasias and within these neoplasias.

Therefore, K-ras mutation and the loss of heterozygosity of microsatellite markers of PaM were investigated in this study, and the current study is thought to be the first report focusing on this.

Our results showed that K-ras mutation was detected in fifteen regions among 52 PaM (28.9%), and LOH-MS was detected in twenty-one regions among 52 PaM (40.4%).

Namely, K-ras mutation and LOH-MS of PaM were not rare and the frequency of those of PaM were higher than those of the normal colonic mucosa, and it came to light that some PaM had the genetic abnormalities which had a relationship to the development of colorectal cancer.

Paneth cells, which are usually situated at the base of the glands of the small intestine, were first identified by Scwalbe [15] in 1872, were studied in detail morphologically by Paneth [16] in 1888.

Now, these cells have been one of the most famous cells in the gastro-intestinal tract, however, the detail function of these is not clear. Paneth cells are sometimes seen in the colorectal tubules, for example in the proximal colonic mucosa of elderly subjects [7,17], in patients with ulcerative colitis [18] and colonic epithelial neoplasia [1,19,20], although the reasons why Paneth cells appear in the large bowel is still unknown.

Described above, many riddles about Paneth cell are still remained. And the gene abnormalities of colonic mucosa with PaM in the current study may be not equal to those of single Paneth cell in the colonic mucosa, because it is difficult to obtain only single Paneth cell in the colonic mucosa, even if microdissection method is used.

However, we have been able to investigate the colonic mucosa with PaM, and we think it very interesting that some PaM have K-ras mutation and the loss of heterozygosity of microsatellite markers, and these PaM may be thought to be the pre-neoplastic mucosa in development of the colonic epithelial neoplasia. Further molecular studies concerning Paneth cell metaplasia in the large bowel should be warranted.

## Conclusions

Colonic mucosa with Paneth cell metaplasia may be one of the pre-neoplastic mucosa in the development of the colonic epithelial neoplasia.

## Abbreviations

PaM, colonic Paneth cell metaplasia; K-ras, K-ras codon 12 mutations; LOH-MS, loss of heterozygosity of microsatellite markers; HE, hematoxylin and eosin; PCR, polymerase chain reaction; ELMA, enzyme linked mini-sequence assay
